# Cytological, physiological and transcriptomic analysis of variegated Leaves in *Primulina pungentisepala* offspring

**DOI:** 10.1186/s12870-022-03808-1

**Published:** 2022-09-01

**Authors:** Jiancun Chen, Yueya Li, Dong He, Meng Bai, Bo Li, Qixiang Zhang, Le Luo

**Affiliations:** Beijing Key Laboratory of Ornamental Plants Germplasm Innovation & Molecular Breeding, National Engineering Research Center for Floriculture, Beijing Laboratory of Urban and Rural Ecological Environment, Engineering Research Center of Landscape Environment of Ministry of Education, Key Laboratory of Genetics and Breeding in Forest Trees and Ornamental Plants of Ministry of Education, School of Landscape Architecture, Beijing Forestry University, 35 Tsinghua East Road, Beijing, 100083 China

**Keywords:** *Primulina pungentisepala*, Leaf variegation, Air space, Chlorophyll metabolism, Transcriptome

## Abstract

**Background:**

*Primulina pungentisepala* is suitable for use as a potted plant because of its beautiful leaf variegation, which is significantly different in its selfed offspring. However, the mechanism of *P.* pungentisepala leaf variegation is unclear. In this study, two types of offspring showing the greatest differences were compared in terms of leaf structure, chlorophyll contents, chlorophyll fluorescence parameters and transcriptomes to provide a reference for studying the molecular mechanism of structural leaf variegation.

**Results:**

Air spaces were found between water storage tissue, and the palisade tissue cells were spherical in the white type. The content of chlorophyll a and total chlorophyll (chlorophyll a + b) was significantly lower in the white type, but there were no significant differences in the content of chlorophyll b, chlorophyll a/b or chlorophyll fluorescence parameters between the white and green types. We performed transcriptomic sequencing to identify differentially expressed genes (DEGs) involved in cell division and differentiation, chlorophyll metabolism and photosynthesis. Among these genes, the expression of the cell division- and differentiation-related leucine-rich repeat receptor-like kinases (LRR-RLKs), xyloglucan endotransglycosylase/hydrolase (*XET/H*), pectinesterase (*PE*), expansin (*EXP*), cellulose synthase-like (*CSL*), *VARIEGATED 3* (*VAR3*), and *ZAT10* genes were downregulated in the white type, which might have promoted the development air spaces and variant palisade cells. Chlorophyll biosynthesis-related hydroxymethylbilane synthase (*HEMC*) and the H subunit of magnesium chelatase (*CHLH*) were downregulated, while chlorophyll degradation-related chlorophyllase-2 (*CHL2*) was upregulated in the white type, which might have led to lower chlorophyll accumulation.

**Conclusion:**

Leaf variegation in *P. pungentisepala* was caused by a combination of mechanisms involving structural variegation and low chlorophyll levels. Our research provides significant insights into the molecular mechanisms of structural leaf variegation.

**Supplementary Information:**

The online version contains supplementary material available at 10.1186/s12870-022-03808-1.

## Introduction

Hara [1] and Sheue et al. [2] proposed that variegated leaves are defined by different colour patterns on the leaf surface, which form regular patterns or irregular spots or patches. Variegated plants are usually herbaceous or climbing plants, with few shrubs or trees, and are mainly distributed in evergreen broad-leaved forests, among which the species diversity is the highest under the shade of tropical rainforests [3, 4]. Variegated plants usually show high shade tolerance and are suitable for displaying indoors as ornamentals. Zhang et al. [5] classified variegated leaves into 5 types: the chlorophyll, pigment, epidermis, air space and appendage types. The chlorophyll and pigment types can be collectively called pigment-related variegation category, while the epidermis, air space and appendage types can be combined into a structural variegation category. However, the mechanisms of leaf variegation is not always composed of a single programme and, in contrast, comprises multiple mechanisms [6].

The formation of leaf variegation has biological significance and is beneficial to plant survival. Some studies have shown that leaf variegation plays important roles in plant adaptation to abiotic factors in the environment, predation prevention, and enhances reproduction. The high photosynthetic efficiency in the variegated leaves of *Arum italicum* was found to be closely related to the palisade tissue structure, which might be a result of the long-term adaptation of this plant to the low light environment under shade [7]. Stehlik et al. [8] suggested that various foliar patterns formed by structural variegation might provide protection against herbivores. Moreover, the leaf variegation of *Silybum marianum* has been shown to provide thermal benefits in cold weather [9].

Mutations of nuclear variegation-related genes were identified in *Arabidopsis* 40 years ago; these mutants include *immutans* (*im*), *variegated 1* (*var1*), *variegated 2* (*var2*) and *variegated 3* (*var3*) [10]. This discovery contributed to a deeper understanding of the leaf variegation formation mechanism [11]. At present, most plants, including crops, fruits, vegetables, and ornamental plants with variegated leaves, are classified into the pigment-related category, for which the molecular mechanism is relatively clear, consisting of 3 main mechanisms: blocked chlorophyll synthesis, chlorophyll degradation, and incomplete chloroplast development. However, structural variegation is mainly related to air spaces and abnormal development of palisade tissue cells. In terms of air spaces, Grimbly [12] suggested that the loose structure between layers L1 (epidermal cells and water-storage cells) and L2 (green tissue) might be caused by slow cell division. Ishizaki [13] pointed out that some transmembrane proteins, such as LRR-RLKs, can regulate cell division, leading to the formation of intercellular spaces [14]. Cell walls are dynamic structures which play key roles in plant cell growth and development [15]. Genes related to cell wall structure and function including *XET/H* [16], *PE* [17], *EXP* [18], and *CSL* [19] can regulate cell wall loosening and stiffening. In terms of the development of palisade tissue cells, mutants of this type mainly present with reduced palisade tissue and spongy tissue cells, especially irregular shaped and incompletely developed palisade tissue cells. For example, mutations in genes including *Variegated and Distorted Leaf* (*VDL*) [20], *Defective Chloroplasts and Leaves* (*DCL*) [21], *VAR3* [22] and *ZAT10* [23] might be the origins of white leaf mutations in tomato, tobacco and *Arabidopsis*, among which the palisade tissue was atypical, with the shape similar to that of spongy tissue.

*P. pungentisepala* is suitable for use as a potted plant because of its morphological characteristics, especially its beautiful leaf variegation, which is significantly different from that of its selfed offspring. Thus, the selfed offspring of *P. pungentisepala* make ideal subjects for better understanding the mechanism of leaf variegation formation in *P. pungentisepala*. Hara and Sheue’s [1, 2] classic study has laid the foundation for the study of structural leaf variegation. In the past 10 years, more studies on the formation of structural leaf variegation have been reported, but these studies did not include the molecular mechanism involved in structural leaf variegation. To better understand the mechanism of leaf variegation formation, transcriptomic analysis was added to the basic research into the optical characteristics of leaves, leaf anatomy, chloroplast ultrastructure, chlorophyll contents and chlorophyll fluorescence parameters.

## Results

### Optical characteristics of the Leaves

**Fig. 1:**
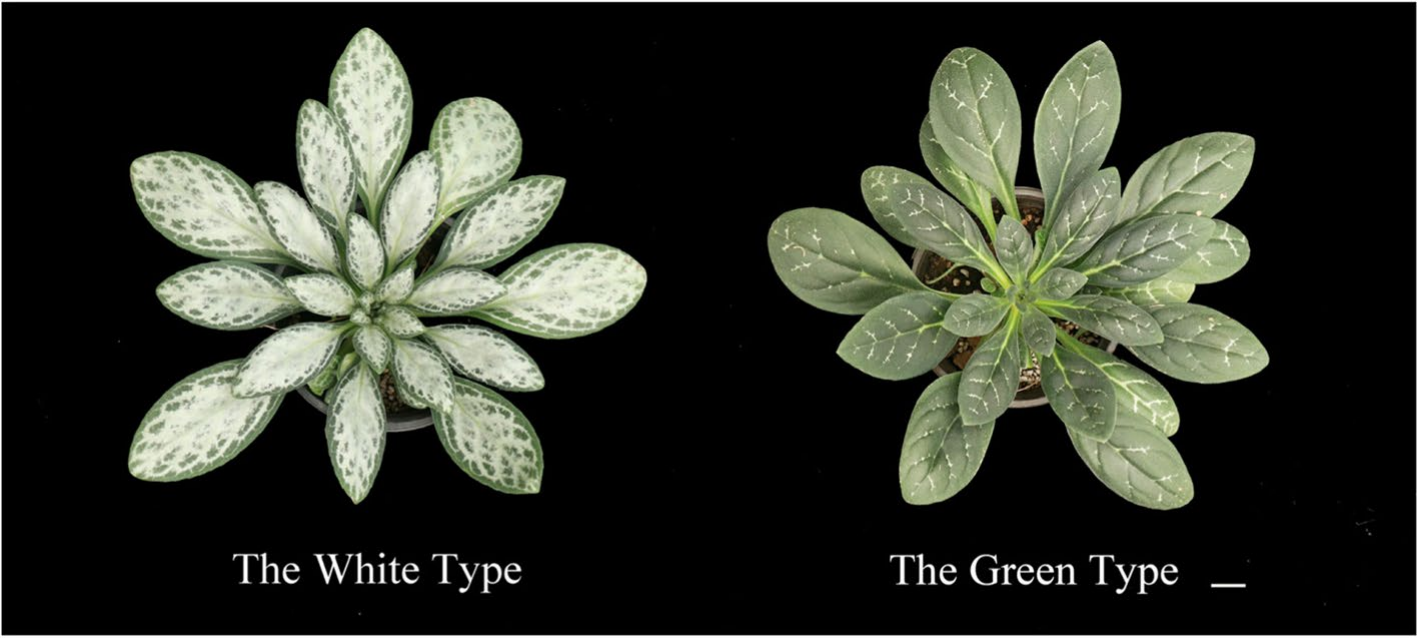
The white type and green type. Scale bar = 2.5 cm

The selfed offspring of *P. pungentisepala* present with different types of leaf variegation. The white type and green type show the greatest difference in the proportion of variegated area, accounting for 70-100% and 0–10% respectively (Fig. 1).

**Fig. 2:**
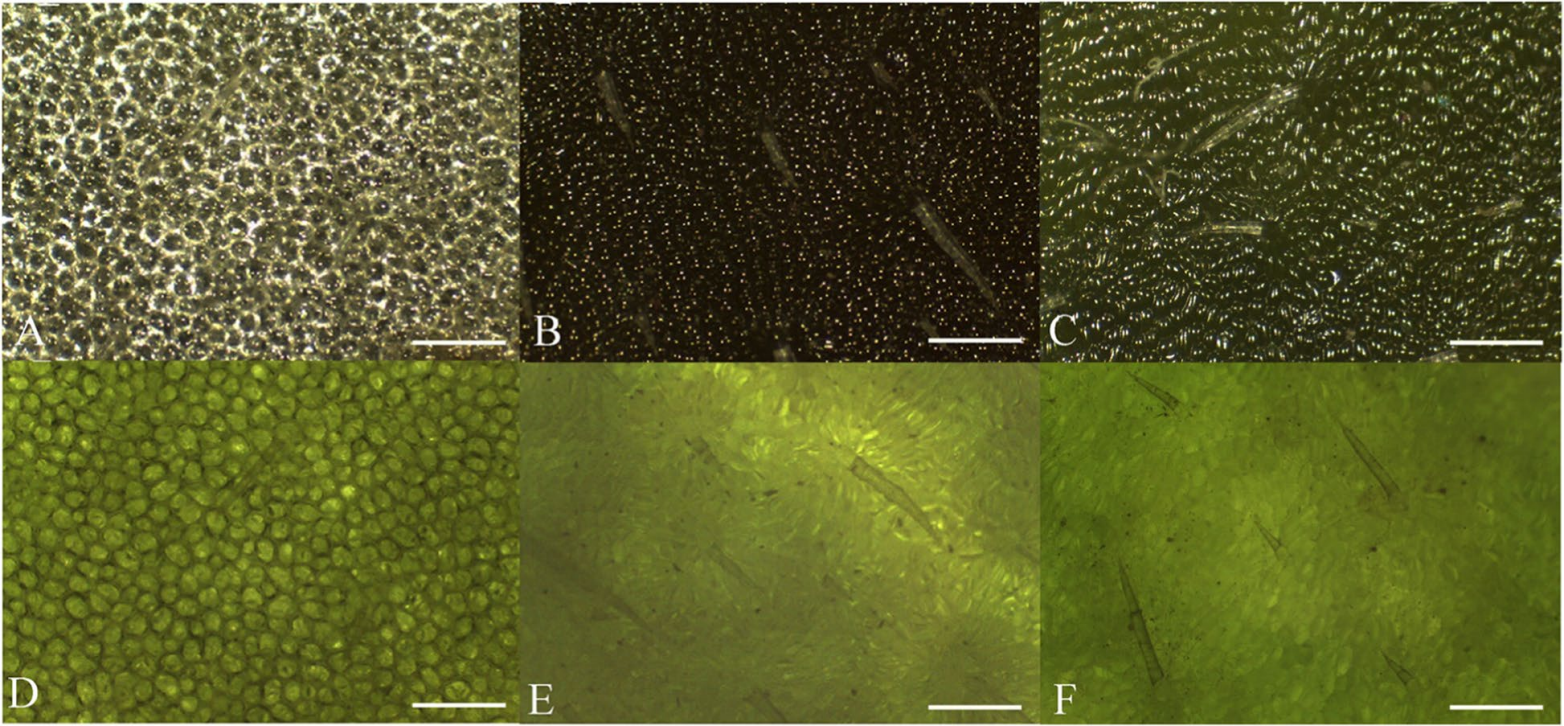
Adaxial surface patterns of leaves of the white type (**A**, **D**) and the green type (**B**, **E**) and the leaves of the white type without air (**C**, **F**) under reflected light (**A-C**) and transmitted light (**D-F**). Scale bars = 0.5 mm

Under reflected light, the white type (Fig. 2 A) showed 2 different light reflection patterns: the spotted pattern (SP) and the polygonal pattern (PP). The SP manifests as white spots formed in the centre of epidermal cells, and the PP manifests as irregular white rings formed around epidermal cell edges. However, in the green type (Fig. 2B), only the SP was found. Under transmitted light, an irregular black ring outlining the epidermal cell edges is seen in the white type (Fig. 2D), while the cell edges are not very clear in the green type (Fig. 2E). After air removal, the differences of the PP and cell edges in the white type compared to those observed in the green type (Fig. 2 C, F) were diminished. Figure S1 shows more details of Fig. 2.

### Anatomical characteristics of the leaves

Leaf width of the white type and green type were measured. According to Table 1, there was no significant difference in leaf width between the two types.

**Table 1:** The leaf width of the white type and green type

	W	G
Width of Leaf / mm	1.41 ± 0.04^a^	1.36 ± 0.32^a^

Values were presented as the mean ± SE of 9 biological replicates. The independent-samples T test was used for comparison analysis. Same superscript a denotes no statistically significant differences (*p* > 0.05). *W* The white type, *G* The green type

**Fig. 3:**
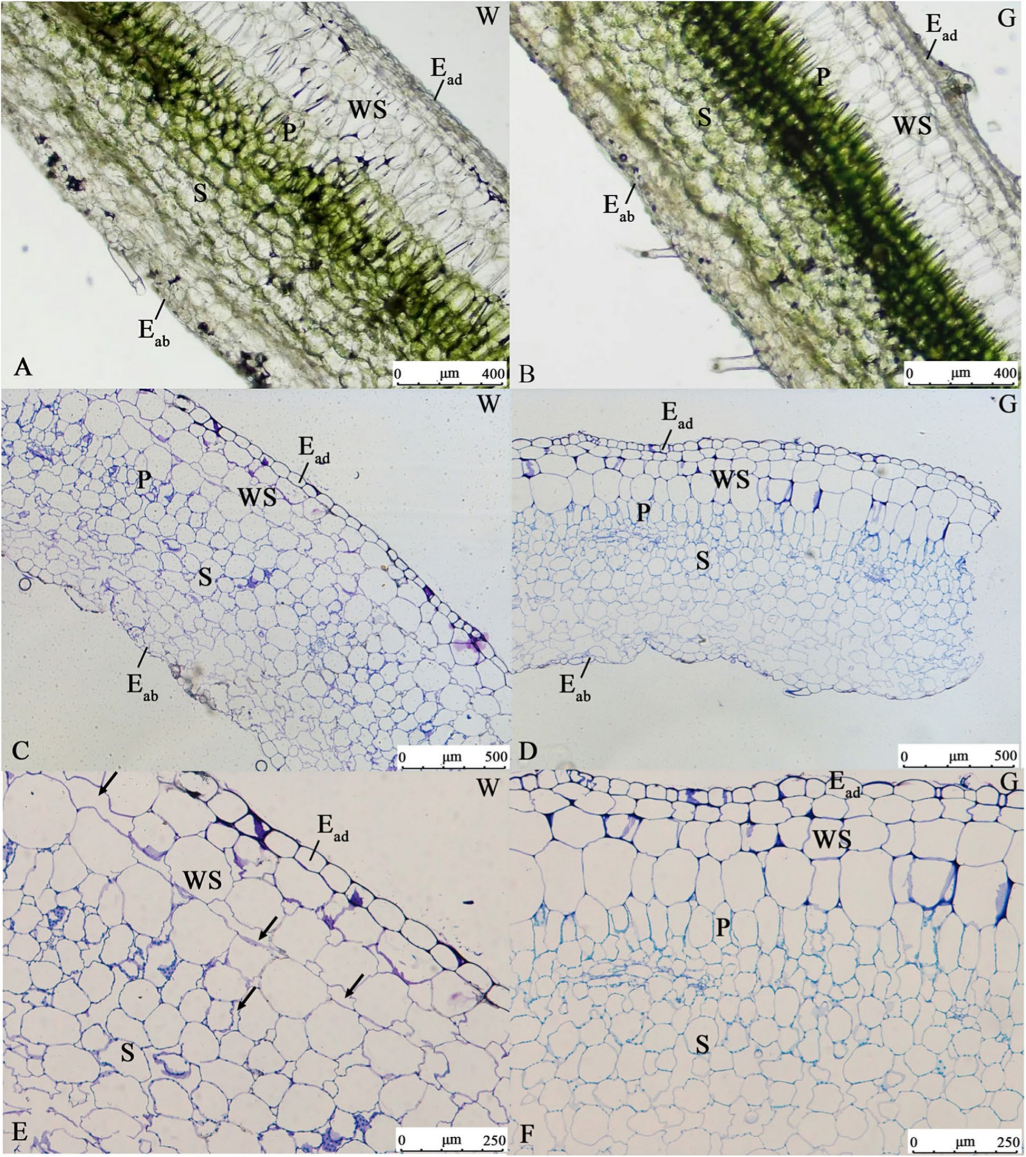
Transverse sections of leaves of the white type (left) and green type (right). (**A**, **B**) White type (**A**) and green type (**B**) leaves prepared by freehand sectioning. (**C-F**) Paraffin sections of white type leaves (**B**, **C**) and green type leaves (**E**, **F**). Arrows: air spaces; W: white type; G: green type; E_ab_: abaxial epidermis; E_ad_: adaxial epidermis; WS: water storage tissue; P: palisade tissue; S: spongy tissue. Scale bars (**A-B**) = 400 μm; (**C-D**) = 500 μm; (**E-F**) = 250 μm

Traverse sections of the white type and green type leaves were prepared. These sections revealed that the epidermal cells of both types formed one layer, and the water storage tissue was composed of three layers, occupying approximately 1/3 of the whole leaf thickness (Fig. 3 A-B). Chlorenchyma cells were found below the water storage tissue, and the green type was darker. In the white type (Fig. 3 C, E), the air spaces were typically found among water storage tissue and palisade tissue, while in the green type (Fig. 3D, F), the water storage tissue and palisade tissue cells were closely arranged. The air spaces, however, were not the only anatomical features distinguishing the white and green types: the shape of the palisade tissue was also obviously different. The palisade tissue was differentiated normally with arranged cylindrical cells in the green type. However, in the white type, some of the palisade tissue cells were spherical and loose, similar to spongy tissue cells.

### Ultrastructure of chloroplasts

**Fig. 4:**
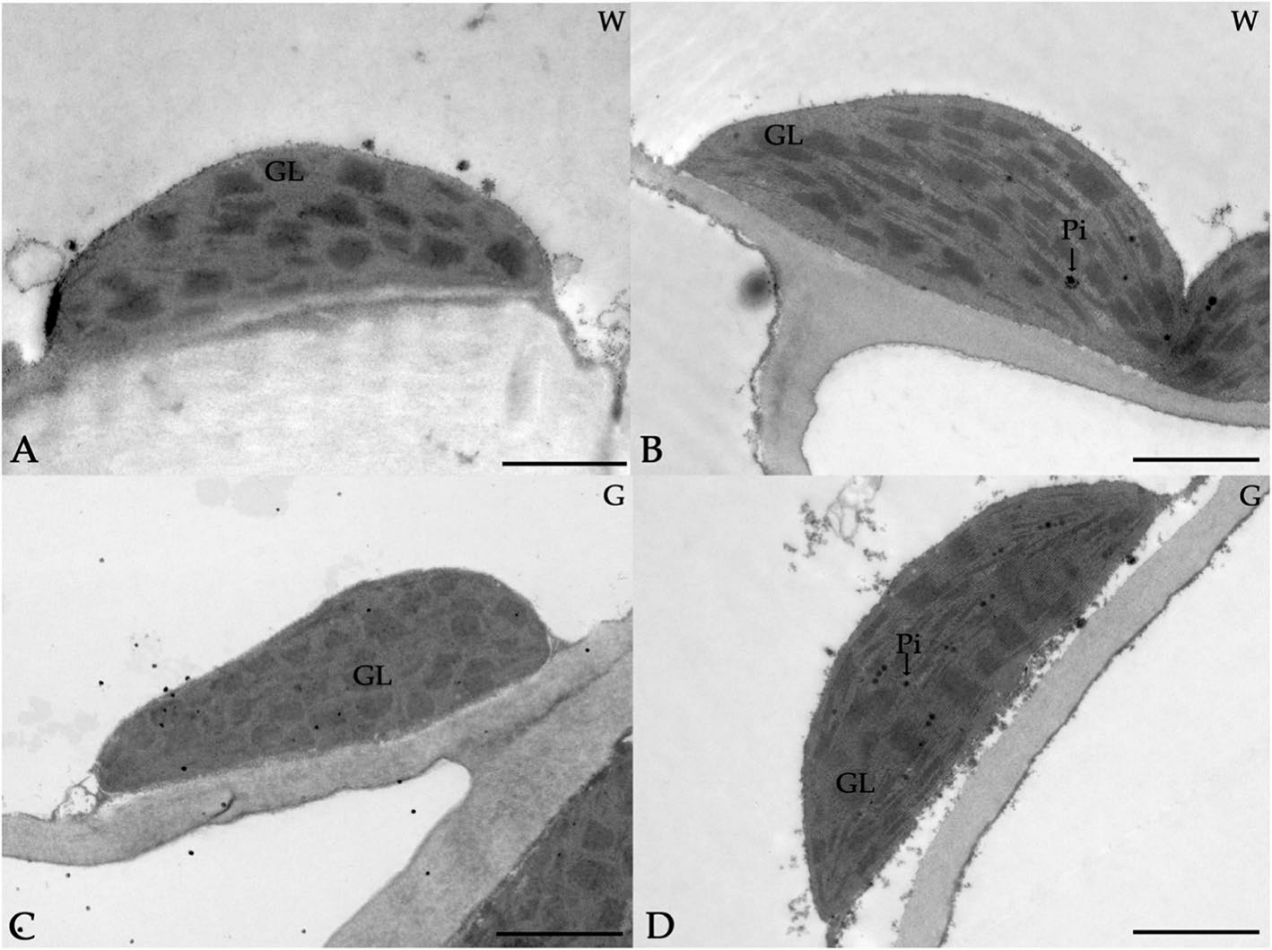
Ultrastructure of the chloroplasts in white type (**A-B**) and green type (**C-D**) leaves. W: white type; G: green type; GL: grana lamella; Pi: plastoglobuli. Scale bar = 2 μm

According to the transmission electron microscope (TEM) observations (Fig. 4), the ultrastructure of the chloroplasts in the white and green types were similar, with both types showing abundant thylakoid membranes and dense grana stacking. Some plastoglobuli were apparent in the sections.

According to Table 2, there was no significant difference in chloroplast size and chloroplasts number per cell, but the cell density of palisade tissue was significantly lower in the white type than that of the green type.

**Table 2:** The size and density of chloroplasts

	W	G
Chloroplasts number/ cell	11.60 ± 2.67^a^	11.30 ± 2.79^a^
Palisade cell number/ mm²	156.69 ± 17.16^b^	223.33 ± 21.46^a^
Chloroplasts size/ um²	16.07 ± 1.85^a^	15.30 ± 2.9^a^

Values were presented as the mean ± SE of 10 replicates. The independent-samples T test was used for comparison analysis. Superscript a and b denote statistically significant differences (*p* < 0.05) in the corresponding parameters between the white type and green type. *W* The white type, *G* The green type

### The chlorophyll contents and chlorophyll fluorescence parameters

Table 3 shows the chlorophyll levels and chlorophyll fluorescence parameters in the white type and green type. The contents of chlorophyll a and total chlorophyll (chlorophyll *a + b*) in the green type were significantly higher than those in the white type. The chlorophyll *b* and chlorophyll *a/b* contents in the white and green types were not significantly different. For the chlorophyll fluorescence parameters, no significant differences in the maximum photochemical quantum yield of the photosystem II (PSII) (Fv/Fm) or effective photochemical quantum yield of PS II [Y(PSII)] were found.

**Table 3:** Chlorophyll content and chlorophyll fluorescence parameters in the white type and green types

	W	G
Chl *a* (mg·g^− 1^ FW)	0.27 ± 0.02^b^	0.47 ± 0.06^a^
Chl *b* (mg·g^− 1^ FW)	0.14 ± 0.04^a^	0.20 ± 0.05^a^
Chl *a + b* (mg·g^− 1^ FW)	0.42 ± 0.06^b^	0.68 ± 0.11^a^
Chl *a/b*	2.00 ± 0.42^a^	2.36 ± 0.34^a^
Fv/Fm	0.763 ± 0.010^a^	0.765 ± 0.003^a^
Y(PSII)	0.490 ± 0.027^a^	0.456 ± 0.032^a^

Values are presented as the mean ± SE of 3 replicates. The independent-samples T test was used for comparison analysis. Superscript a and b denote statistically significant differences (*p* < 0.05) in the corresponding parameters between the white type and green type. *Chl a* Chlorophyll a, *Chl b* Chlorophyll b, *Chl a+b* Chlorophyll a+b, *Chl a/b* The ratio of chlorophyll a to chlorophyll b, *Fv/Fm* Maximum photochemical quantum yield of PS, *Y(PSII)* Effective photochemical quantum yield of PS II, *FW* Fresh weight, *W* The white type, *G* The green type

### Transcriptomic analysis

The leaves of the white type and green type were used for RNA-Seq analysis, and a total of 6 samples (3 biological replicates for each type) were sequenced. After the low-quality reads were removed, 93,827 unigenes were identified with an average length of 554 bp. The length distribution of the unigenes is presented in Fig. S2. An overview of the RNA-Seq data can be seen in Table S1.

### DEGs in different type of leaves

A total of 2880 DEGs were identified in the green type vs. white type, among which 1131 genes (39.27%) were upregulated and 1749 genes (60.79%) were downregulated. Cluster analysis and volcano plots of DEGs are displayed in Figs. S3 and S4. Subsequently, more functional information on the DEGs was obtained through Gene Ontology (GO) and Kyoto Encyclopedia of Genes and Genomes (KEGG) enrichment analysis.

**Fig. 5:**
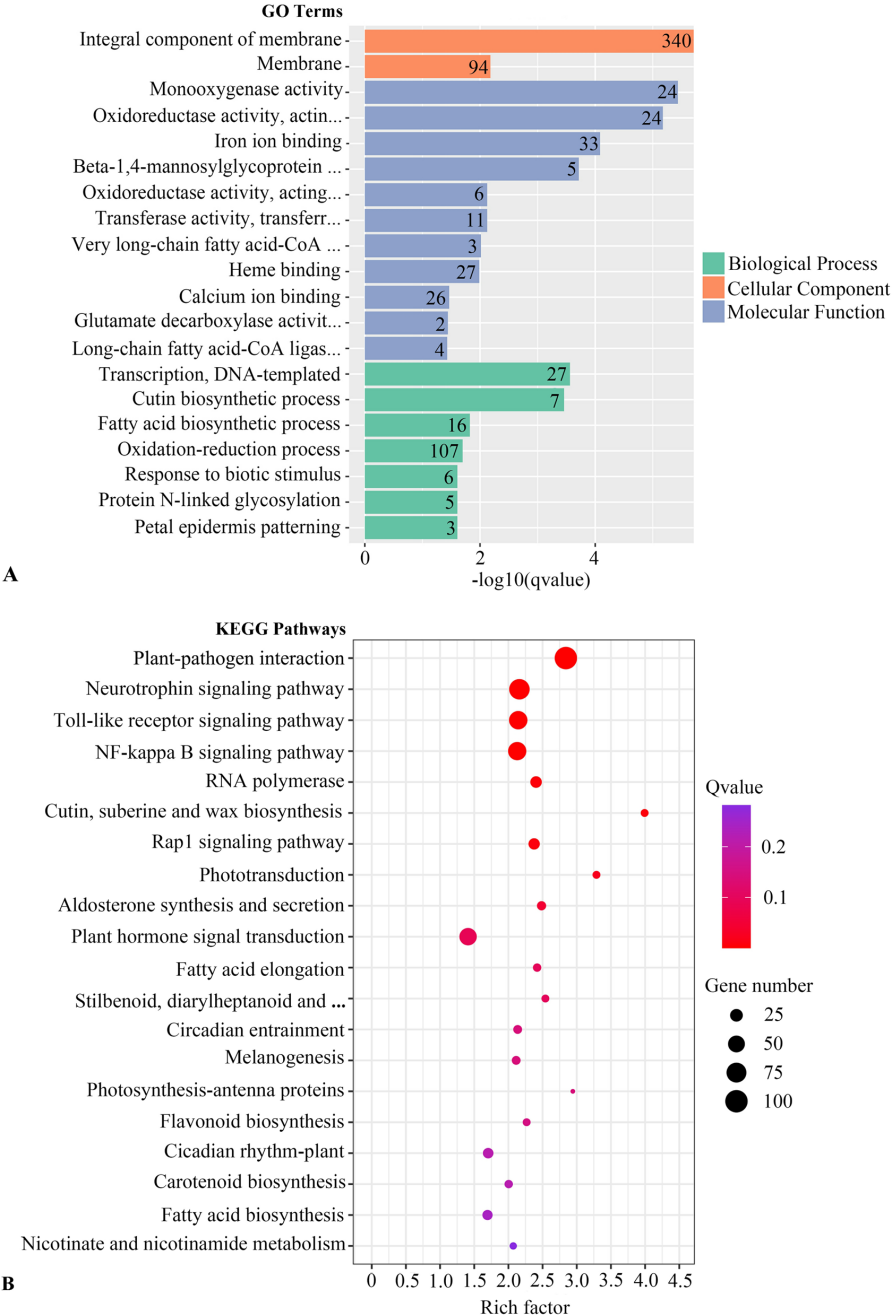
The 20 GO terms and KEGG pathways most enriched with DEGs. **A** GO term enrichment; **B** KEGG pathway enrichment

Based on the GO classification (Fig. 5 A), the DEGs could be assigned to 3 major GO categories: CC (cellular component), MF (molecular function) and BP (biological process). Specifically, 15 GO classes were identified in the CC category, 15 GO classes were identified in the MF category, and 24 classes were identified in the BP category. The analysis of transverse leaf sections indicated that air spaces and the shape of the palisade cells played a vital role in the development of variegated leaves; therefore, we focused on cell development and division, which are related to the plasma membrane. In the CC category, ‘integral component of membrane’ (GO: 0016021) and ‘membrane’ (GO: 0016020) were significantly enriched. Specifically, a total of 340 DEGs were identified with ‘integral component of membrane’, among which 224 DEGs were downregulated and 116 DEGs were upregulated. In ‘membrane’, the number of enriching DEGs was 94, which included 73 downregulated DEGs and 21 upregulated DEGs. Cell development and division also largely depend on the cell wall loosening and stiffening. In ‘cell wall’, the number of enriching DEGs was 20, which included 16 downregulated DEGs and 4 upregulated DEGs.

To investigate the specific pathways related to the formation of leaf variegation, the DEGs were subjected to a KEGG pathway enrichment analysis (Fig. 5B). The DEGs fell into 285 pathways in the KEGG database. ‘Photosynthesis-antenna proteins’ (ko00196) and ‘phototransduction’ (ko04744) were both identified among the 20 most enriched KEGG pathways, and 6 DEGs and 9 DEGs were involved with these 2 pathways, respectively. Because the chlorophyll content of the white type was lower than that in the green type, more attention should be directed to pathways related to chlorophyll metabolism. Seven DEGs were identified in the ‘porphyrin and chlorophyll metabolism’ (ko00860) pathways.

### Genes involved in cell development and division

**Fig. 6:**
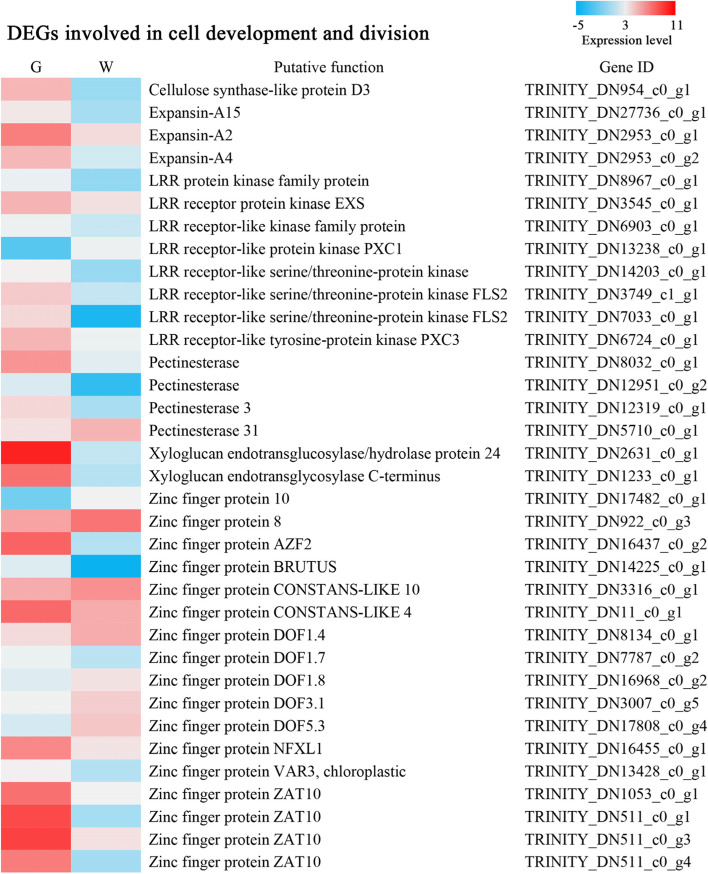
DEGs involved in cell development and division. W: white type; G: green type. The expression levels of each gene (log2 FPKM) in the white type and green type are indicated by red and blue rectangles, with the red rectangles representing upregulated gene expression, and blue rectangles representing downregulated gene expression

**Fig. 7:**
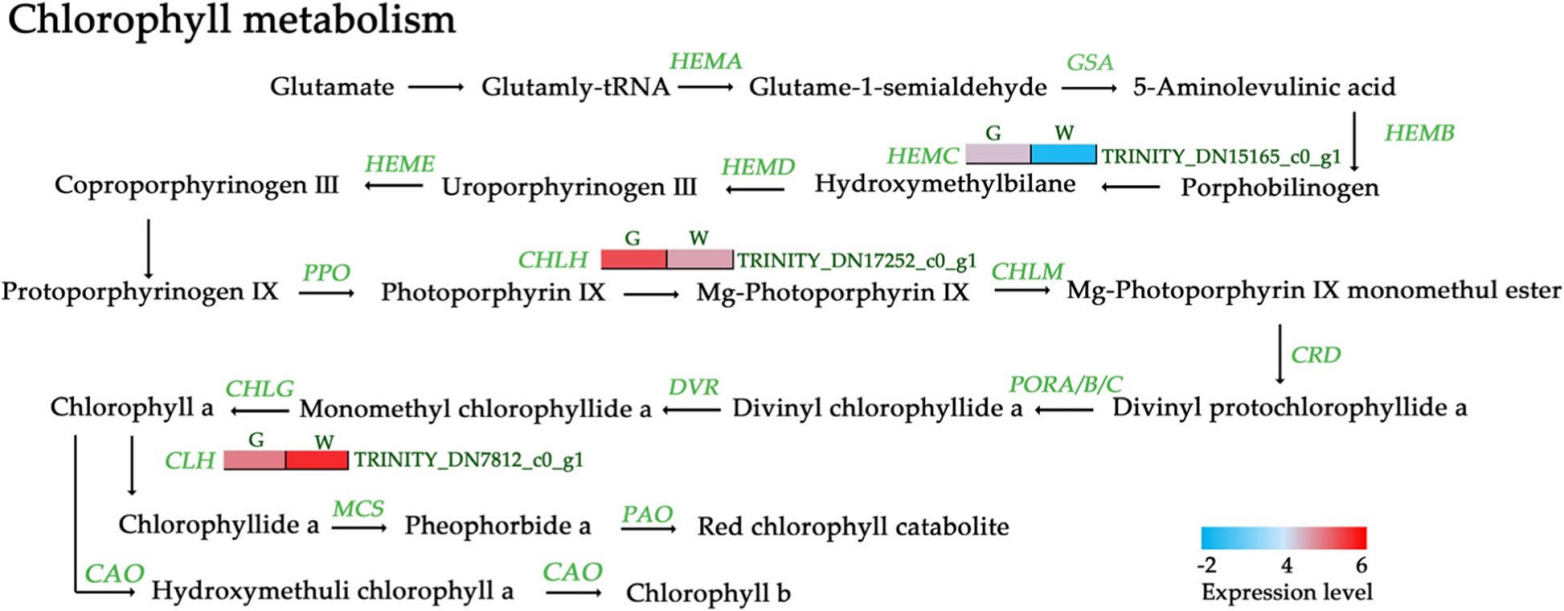
DEGs involved in chlorophyll metabolism. W: the white type; G: the green type. The expression levels of each gene (log2 FPKM) in the white type and green type are indicated by red/blue rectangles. Red rectangles represent upregulated genes, and blue rectangles represent downregulated genes

A GO enrichment analysis revealed that DEGs in ‘integral component of membrane’ and ‘membrane’ were dominant, and some key functional genes affected cell development and division. LRR-RLKs play important roles in the regulation of cell wall remodelling during cell division [13]. According to Figs. 6 and 7 DEGs encoding LRR-RLKs were downregulated, and 1 DEG was upregulated. The plant cell wall is a dynamic structure that is the foundation of plant growth and development [24]. In our study, 10 DEGs belonging to the *XET/H*, *PE*, *EXP*, and *CSL* gene families were all downregulated.

Zinc finger proteins play a key role in the regulation of cell differentiation, and the zinc finger proteins *VAR3 and ZAT10* have been previously shown to regulate palisade cell development [17, 18]. In our transcriptomic analyses, 1 DEG identified as zinc finger protein *VAR3* and 3 *DEGs* encoding *ZAT10* were downregulated. These DEGs might promote the variation in the shape of palisade cells.

### Genes related to chlorophyll metabolism and photosynthesis

*CHLH* insertion of Mg^2+^ into protoporphyrin IX [25] and chlorophyll degradation are vital processes in the chlorophyll metabolism pathway. The KEGG enrichment analysis revealed that 7 DEGs were closely related to porphyrin and chlorophyll metabolism, and 3 of these DEGs were involved in chlorophyll metabolism (Fig. 7). Specifically, 1 DEG encoding *CHL2* was upregulated, and 2 DEGs encoding *CHLH* and *HEMC* were downregulated; all these DEGs contributed to a decrease in chlorophyll levels.

**Fig. 8:**
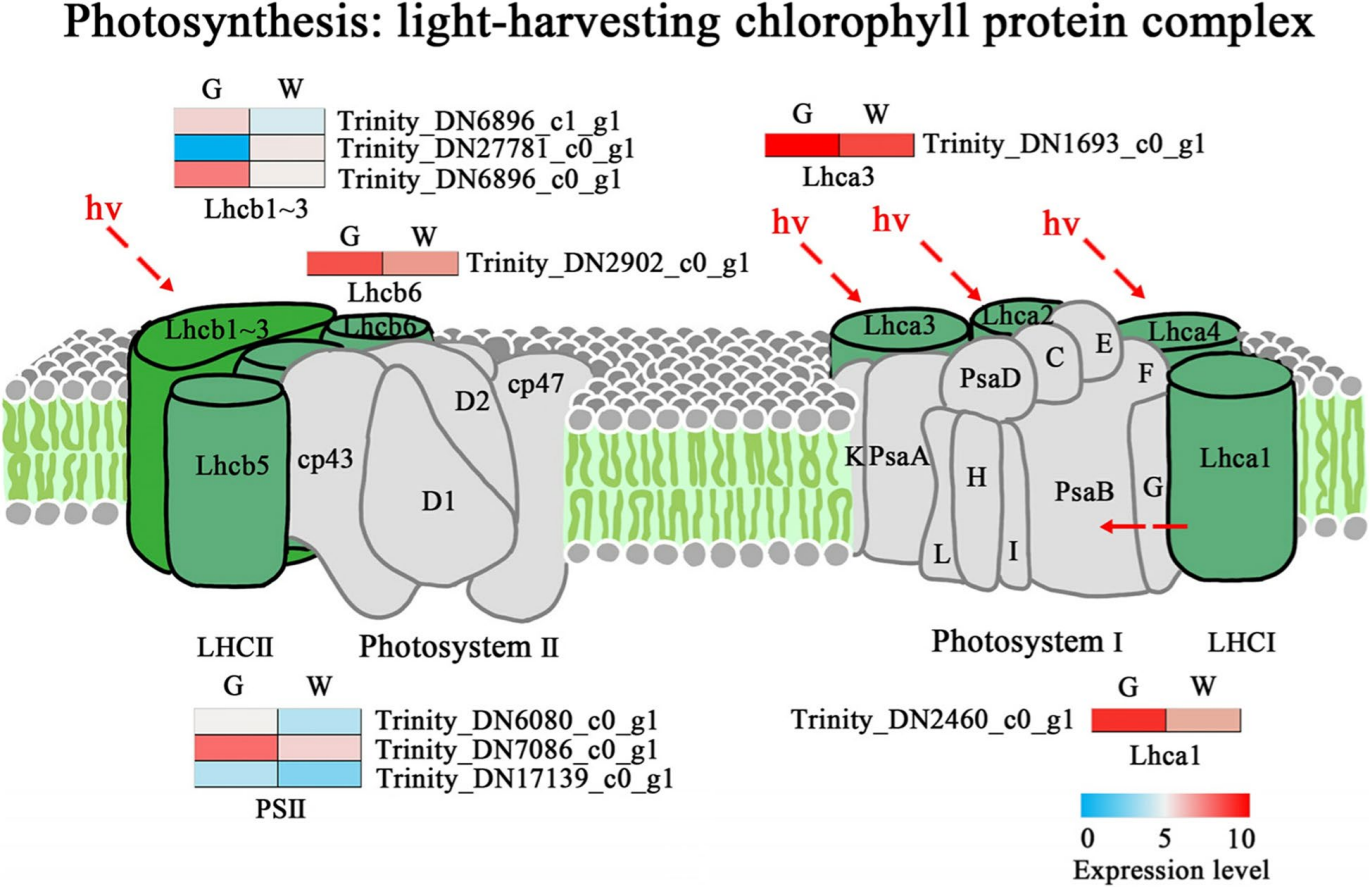
DEGs involved in the photosynthesis-annotated proteins (ko00196) [26]. W: white type; G: green type. The expression levels of each gene (log2 FPKM) in the white type and green type are indicated by red/green rectangles, with the red rectangles representing upregulated gene expression, and blue rectangles representing downregulated gene expression

In the pathway of photosynthesis-antenna proteins (Fig. 8), the light-harvesting chlorophyll protein complex was differentially expressed between the white type and green type. Six DEGs encoded chlorophyll a-b binding proteins, among which 1 DEG was upregulated and 5 DEGs were downregulated. Three DEGs related to PS II were all downregulated.

### Verification of RNA sequencing data by qRT‒PCR

**Fig. 9:**
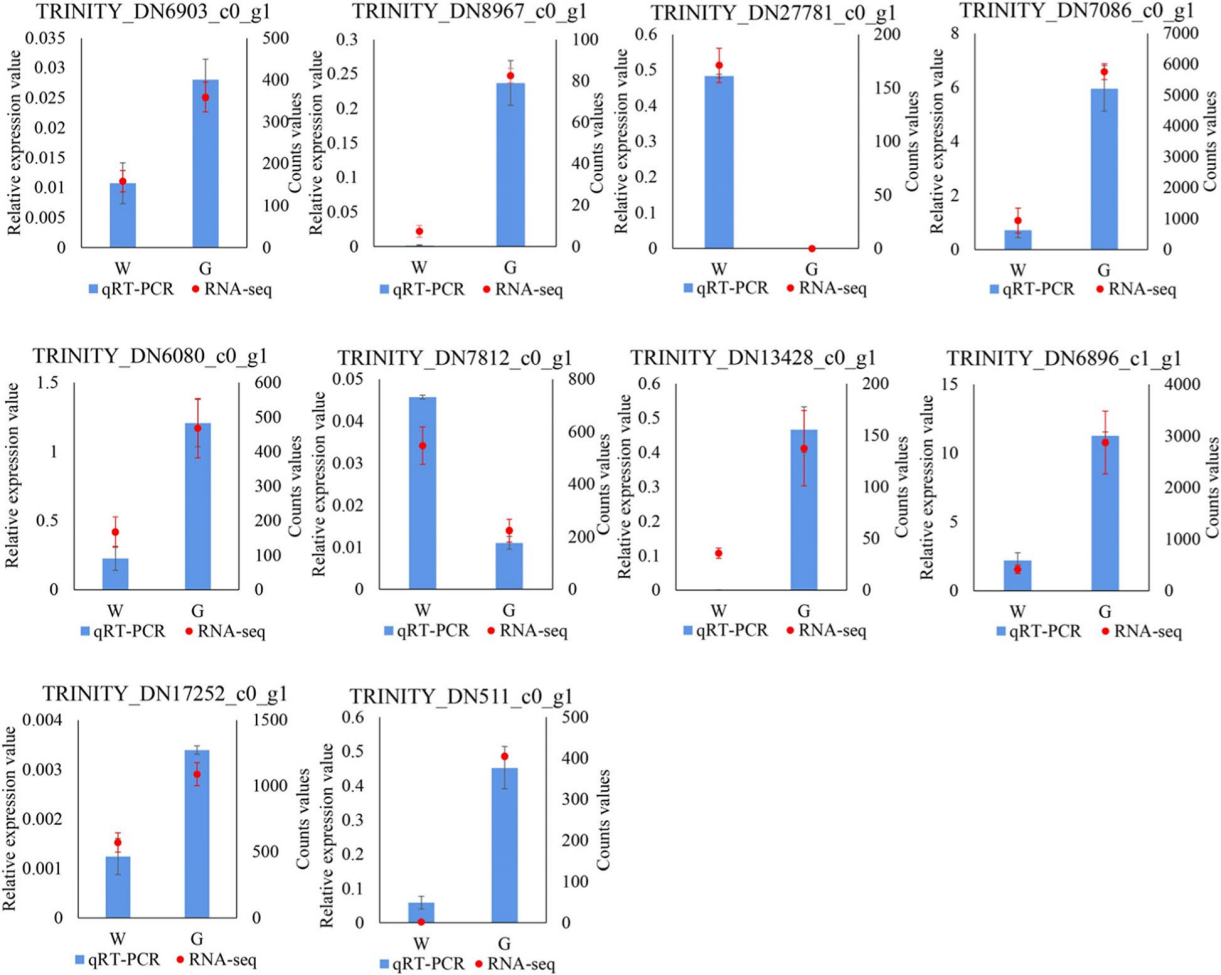
qRT‒PCR validation of 10 genes randomly selected from DEGs encoding proteins involved in cell development and division, chlorophyll metabolism and photosynthesis. The data were normalized to the reference gene *Actin7*. The data are presented as the mean ± SE. Three biological replicates and 3 technical replicates were established for each gene. W: white type; G: green type

To validate the RNA-Seq results, 10 DEGs were selected and subjected to qRT‒PCR analysis with *Actin7* used as an internal reference gene (Fig. 9). Four DEGs were shown to be related to cell development and division, including LRR-RLKs and *VAR3*. Six DEGs were shown to be related to chlorophyll metabolism and photosynthesis, such as chlorophyll a-b binding protein, psbP domain-containing protein, PS II 22 kDa protein and *CHL2*. The qRT‒PCR results showed that 8 DEGs were downregulated and 2 were upregulated. These results were generally consistent with the RNA-Seq data, indicating that the transcriptome data were reliable.

## Discussion

Hara [1] indicated that many plant genera belong to the ‘air space’ variegation type, such as *Arisaema*, *Begonia*, *Clematis*, *Cyclamen*, *Ornithogalum*, *Pyrola*, *Saxifraga* and *Viola*. The location of the air spaces varies from plant to plant, with some found between the adaxial epidermal cells and the upper mesophyll, such as in *Sonerila heterostemon* [27], *Begonia formosana* [2] and *Nervilia nipponica* [28]; others surrounding spongy tissue [7]; and others located between the chlorenchyma and the water storage tissue, as in a *Begonia* cultivar [2]. In our study, air spaces were identified between the water storage tissue in the white type, which had not been observed in previous studies.

Leaf transverse sectioning is not the only way to prepare plants to observe air spaces. A PP is a unique feature of the variegation in *Begonia* [29], and it is composed of irregular white rings around the adaxial epidermal cell edges, which can be viewed under reflected light. This phenomenon has also been seen in the white type. Proving the presence of air spaces, the PP in the white type disappeared when the air space was replaced with water. After the air was replaced, no difference was observed between the white type and green type under reflected or transmitted light.

Differences in air space are not the only difference between these two types. Air space variegation is related to variations in palisade-cell development. In *Begonia*, the typical funnel-shaped chlorenchyma appears isodiametric in light areas [2]. In pale-green sectors of *A. italicum* leaf, the palisade tissue is reduced to a loose layer that is half the thickness of that in dark-green sectors and is composed of small, underdeveloped cells [7]. In the white area of leaves in *Blastus cochinchinensis* [6], the upper mesophyll is composed of three or four layers of colourless sponge-like cells, which contribute to the higher leaf thickness. In our study, we found that the palisade cells in the green type were tightly arranged and column-shaped, while in the white type, they were rounded, sponge-like cells with air spaces between them. In addition, there was no significant difference in leaf width between the two types.

We found no significant difference in chloroplasts size, chloroplasts number per cell and the ultrastructure of chloroplasts between the white type and green type. Both types presented well-developed grana and entirely filled thylakoid membranes in chloroplasts, but the cell density of palisade tissue was significantly lower in the white type than that of the green type. We further examined the chlorophyll contents. The levels of chlorophyll *a* and chlorophyll *a + b* in the white type were significantly lower than those in the green type. We considered that it might be due to the lower cell density of palisade tissue in the white type. In previous studies of structural variegation, the chlorophyll levels in variegated areas, such as in *B. cochinchinensis* [6], *(A) italicum* [7], *(B) rex* [29], *and Cyclamen persicum* [27], were lower than those in green areas. Rocca [6] suggested that the decreased content of chlorophyll in the variegated area was due to the reduction in layer numbers and size of the palisade tissue cells. However, in *Aglaonema nitidum*, no significant difference was found [30]. The chlorophyll fluorescence parameters in both the white and green types were not significantly different, which indicated that the white type underwent normal photosynthesis. In previous studies, the chlorophyll fluorescence parameters of structural leaf variegation were normal and similar to those in green areas [2, 6, 7]. Aelita [27] pointed out that although some leaves with structural variegation show a low chlorophyll content, photosynthesis proceeds normally. We hypothesized that photosynthesis might have reached saturation at a low chlorophyll content.

RNA-Seq analyses have been widely used to identify DEGs in different developmental stages or under different physiological conditions. Through an RNA-Seq analysis, the mechanisms of leaf variegation in *Ginkgo biloba* [31] and *Ilex × altaclerensis* ‘Belgica Aurea’ [32] were clarified. Thus, RNA-Seq technology and related analysis methods enable the identification of leaf variegation mechanisms. In our study, the Illumina sequencing results led to the identification of 2880 DEGs in the leaves of the white type and green type *P. pungentisepala*. Some DEGs related to the development and growth of cells, chlorophyll metabolism and photosynthesis are likely involved in leaf variegation formation in *P. pungentisepala*.

Air spaces and variant palisade tissue are critical for structural leaf variegation. DEGs known to encode LRR-RLKs play major roles in regulating the development and growth of cells in plants. Interestingly, these DEGs were classified into ‘integral component of membrane’ and ‘membrane’ categories, and approximately 80% of them showed downregulated expression. In our study, 5 DEGs encoding LRR-RLKs were downregulated. In *Arabidopsis thaliana*, LRR-RLKs and *HAESA* regulate floral organ abscission, and the amount of *HAESA* protein is inversely correlated with defective cell separation [33]. The development and division of cells are also closely related to cell wall loosening and stiffening [34]. In our study, the expression of 10 DEGs in the *XET/H*, *PE*, *EXP*, and *CSL* gene families, which are related to cell wall structure, was downregulated. These DEGs might differentially slow cell division in the L1 and L2 layers. In addition, zinc finger proteins, such as *VAR3* and *ZAT10*, play important roles in cell differentiation. Naested [16] suggested that *VAR3* is an important protein required for palisade cell development, and palisade cells fail to expand in the variegated sectors in *var3* [35]. The *Arabidopsis var3* mutant with white leaf variegation and variant palisade tissue was found to be similar to *P. pungentisepala*. *ZAT10* induced the formation of cylindrical palisade tissue cells mainly by controlling the expansion of palisade cell width [23]. In our study, the expression of 1 DEG encoding *VAR3* and 4 DEGs encoding *ZAT10* was downregulated, which might have promoted variegation based on the shape of the palisade cells.

The chlorophyll metabolism pathway plays a key role in mutant leaf variegation and cell senescence [36], and depending on the chlorophyll type, these mutants may present with defective chloroplasts and thus disrupted photosynthesis. In previous studies, leaves with structural variegation were thought to contain functional chloroplasts and perform normal photosynthesis, although the chlorophyll levels were low, but normal for a green leaf [27]. In our study, the contents of chlorophyll *a* and chlorophyll *a + b* were significantly lower in the white type. Moreover, 3 DEGs related to chlorophyll metabolism were associated with a decrease in chlorophyll content, which was consistent with the lower chlorophyll levels observed. Magnesium chelatase is a key factor in the biosynthesis of chlorophyll [37]. A decrease in magnesium chelatase activity results in a decrease in the content of chlorophyll in a leaf variegation mutant [38]. The higher expression of genes involved in the chlorophyll degradation pathway might also result in a decrease in the content of chlorophyll. The expression of *CHL2* in *Cymbidium sinense* ‘Dharma’ mutants was higher, leading to a decrease in chlorophyll content, which might be the reason for its colour difference [39]. In our study, 1 DEG, encoding *CHL2*, was upregulated, which might have led to a decrease in the chlorophyll content. Moreover, the expression of 6 DEGs encoding chlorophyll a-b-binding proteins was decreased in the white type. This might have been due to the decrease in chlorophyll content [31, 40]. However, these changes did not affect the chlorophyll fluorescence parameters. In a previous study, leaf variegation of the chlorophyll type often exhibited both low chlorophyll levels and low chlorophyll fluorescence parameters, which differed from the leaf variegation of *P. pungentisepala*.

In addition, we found cutin-related processes were enriched in GO terms and KEGG pathways, which might be due to the stress resistance. Cutin-related processes are closely related to the stress resistance [41]. We hypothesized that the white type might be significantly different from the green type in stress resistance.

**Fig. 10:**
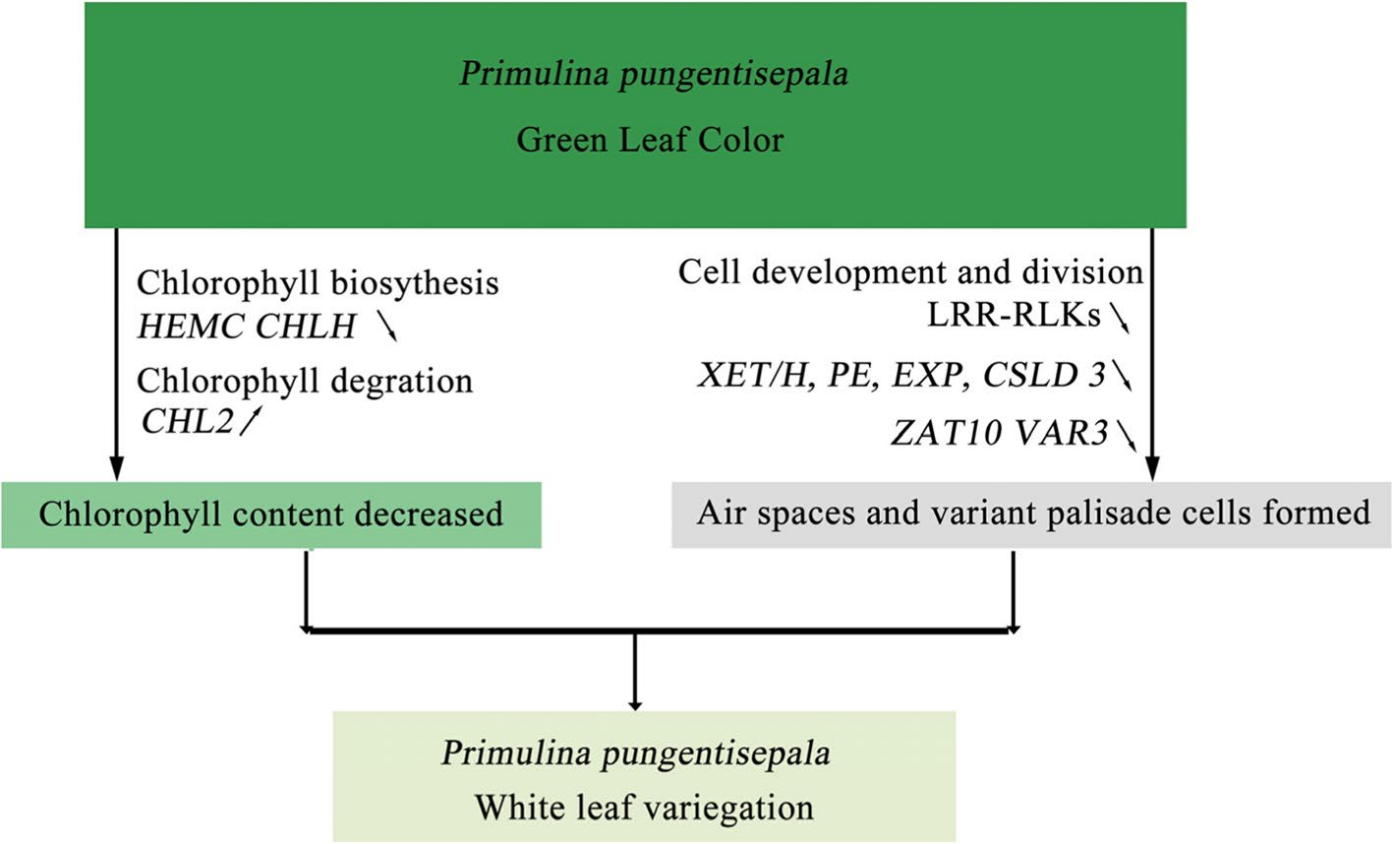
The proposed pathway of leaf variegation in *P. pungentisepala*

In summary, we proposed a pathway that leads to leaf variegation in *P. pungentisepala* (Fig. 10). Changes in the expression of genes related to chlorophyll biosynthesis and degradation lead to reduced chlorophyll accumulation in white leaf variegation. Furthermore, changes in the expression of genes related to cell division and cell differentiation lead to air space formation and variant palisade cell development. These changes might exhibit a close connection with white leaf variegation.

## Conclusion

*P. pungentisepala* is a potential material of potted plants and its beautiful leaf variegation was significantly different in its selfed offspring. Especially the white type, which leaves have silvery metallic luster. In this study, we investigated differences between the white type and green type. Performing a cytological analysis, we found that air spaces were abundant between water storage tissues and the normal ultrastructural characteristics of chloroplasts. Performing a physiological analysis, we found lower chlorophyll levels in the white type; however, these leaves showed the same normal chlorophyll fluorescence parameters as the green type. Therefore, we suggest that the leaf variegation in *P. pungentisepala* is caused by a combination of mechanisms, including structural variegation and lower chlorophyll levels. A transcriptional sequence analysis leads to the identification of DEGs involved in cell division and differentiation. LRR-RLKs are related to membrane; *XET/H*, *PE*, *EXP*, and *CSL* family genes are related to cell walls; and *ZAT10* and *VAR3* are related to cell differentiation; the expression of all these DEGs was downregulated. The downregulation of these genes might have led to air space and variant palisade cell formation. DEGs involved in chlorophyll metabolism and photosynthesis were also identified. The upregulation of *HEMC* and *CHLH* and the downregulation of *CHL2* might have caused a decrease in chlorophyll levels. Thus, these DEGs might have caused white leaf variegation. Moreover, qRT‒PCR verified that these DEGs were differentially expressed between the white type and green type. Our findings provide a reference for the molecular mechanism of structural leaf variegation.

## Methods

### Plant materials

*P. pungentisepala* in the greenhouse were introduced from an area between Guangxi and Beijing and have been domesticated for years. The green type and white type showed the largest differences in variegation area produced in the selfed offspring of *P. pungentisepala* in 2019. All the plant materials were properly maintained in a greenhouse of Beijing Forestry University, which is maintained at an average annual temperature of 20 °C and a relative humidity of 75%. The maximum light intensity was 30,000 lx.

### Optical properties of the leaves

The adaxial surfaces of fresh leaves of the white type and green type and the leaves of the white type without air spaces were observed under both transmitted and reflected light with a LEICA M165FC fluorescent stereo microscope (Wetzlar, Germany).

The leaves of the white type without air spaces were prepared by cutting into 5.0 mm × 5.0 mm leaf discs and then placed in a centrifuge tube with distilled water. The samples were subjected to a vacuum (-40 kPa) for 5 h. The vacuum pump was not turned off until all the disks sank to the bottom [29].

### Leaf structure and chloroplast ultrastructure

Small pieces of leaves (2.0 mm × 2.0 mm) from the white type and green type were prepared. Some of the samples were prepared by freehand sectioning. Some samples were fixed in formaldehyde-acetic acid-ethanol fixative (FAA) for 24–48 h at room temperature. After the tissues were dehydrated in ethanol, they were subjected to xylene for making them transparent and then embedded in paraffin. The samples were cut into paraffin Sect. (8 μm) with a microtome [42]. Other samples were fixed in 2.5% glutaraldehyde overnight at 4 °C. After the tissues were dehydrated in ethanol, postfixed with 1% OsO_4_ and embedded in resin, they were cut into ultrathin Sect. (70 nm) with an MTX ultramicrotome (RMC, Tucson, AZ, USA). The paraffin Sect. (8 μm) were stained with toluidine blue, and the ultrathin Sect. (70 nm) were stained with 5% uranyl acetate (in 50% methanol) and 1% lead citrate (in water). The freehand-prepared and paraffin-embedded sections were observed with an optical microscope (Leica DMC4500, Wetzlar, Germany), and the anatomical characteristics were compared. The ultrathin sections were observed with a transmission electron microscope (JEOLJEM-1400, Tokyo, Japan), and the chloroplast ultrastructures were compared. The chloroplasts in 10 randomly selected cells were counted using the ultrastructural images, and the average number of chloroplasts per cell was calculated. The mean size of chloroplasts in 10 intact chloroplasts distributed in cells was determined using ImageJ. The palisade cells in 10 randomly selected area were counted using the paraffin section images, and the area was calculated using ImageJ [31].

Leaf width of the white type and green type were measured by digital vernier caliper, and the measurement points were at the same position of the leaf [43].

### Measurements of the chlorophyll content and chlorophyll fluorescence parameters

Approximately 0.2 g (fresh weight) of leaf tissue from white leaves and green leaves was placed into separate mortars, and a small amount of quartz sand, calcium carbonate powder and 95% ethanol were added to each mortar, and the leaves were thoroughly ground. After filtration and washing, the solutions were put into a 25-mL volumetric flask with ethanol. The absorbance of ethanol extracts was measured at 665 and 649 nm with a Biomate 3 S UV‒visible spectrophotometer (Thermo Fisher Scientific, USA). The chlorophyll a and b contents were subsequently calculated with the appropriate equations [44].

Chlorophyll fluorescence parameters were measured in the second or third round of leaves of the white type and green type with a PAM-2500 portable amplitude modulation fluorometer (Walz, Germany). The chlorophyll fluorescence parameters were measured after the leaves had been adapted to the dark for 20 min. The F_o_ (the minimal fluorescence value) was determined after dark adaptation, and a 0.8-s saturating light pulse at an intensity of 8000 µmol m^− 2^ s^− 1^ was applied to obtain the F_m_ (the maximal fluorescence). The light intensity was 1000 µmol m^− 2^ s^− 1^ during the measurement of the F_o_’ (the minimal fluorescence at the light-adapted state) and F_m_’ (the light-adapted maximal fluorescence yield) [45].

Fv/Fm and Y(PSII) were calculated according to the appropriate formulas [46, 47].

### RNA‑Seq

For the transcriptome sequencing experiment, plants were grown in a greenhouse with sun-shading nets, and the temperature was approximately 25°C. The innermost leaves of the white and green types were sampled and frozen in liquid nitrogen and then stored at − 80°C. Each sample weighed 0.2 g, and 3 biological replicates were established. Total RNA was isolated from the different samples. The quality and quantity of the RNA were determined with a Nanodrop 1000 Spectrophotometer (Thermo Fisher Scientific, Wilmington, DE) and Agilent 2100 Bioanalyzer (Agilent Technologies, Santa Clara, CA). The mRNAs of each sample were enriched using oligo (dT)-attached magnetic beads. Then, the mRNAs were sheared into short fragments with fragmentation buffer, and first-strand cDNA was synthesized using random hexamers and reverse transcription. Then, buffer, dNTPs and DNA Polymerase I were added to the cDNA to synthesize second-strand cDNA. Subsequently, AMPure XP Beads were used to purify the double-stranded cDNA. The double-stranded cDNA fragments were ligated to an adapter with a ‘T’ at the 3’ end. Then, AMPure XP beads were used for fragment size selection, and PCR enrichment was performed to obtain the final cDNA library. Finally, Illumina sequencing was performed with an Illumina NovaSeq6000 instrument at Beijing Biomics Biotech Co., Ltd. The raw sequencing data have been deposited with the National Center for Biotechnology Information (accession number: PRJNA815489).

### Transcriptome data processing and analysis

We removed adapter sequences, reads with ambiguous bases ‘N’, low-quality reads (Q value < 10) and fragments less than 20 bp in length from the raw data to obtain high-quality clean data. The full-length transcriptome of *P. pungentisepala* was constructed without a reference genome from RNA-Seq data by using Trinity [48].

Gene expression levels were estimated by RSEM for each sample. Differential expression analysis between different samples was performed with DESeq2 [49]. Genes with a fold change ≥ 2 and FDR < 0.01 were considered DEGs. All DEGs were mapped to each term in the GO and KEGG databases.

### qRT‑PCR validation of transcript levels

Ten DEGs were selected from all the DEGs identified to validate the reliability of the RNA-Seq analysis. The primers used for these assays were designed using Primer 5.0 software [50] and are presented in Table S2. qRT‒PCR was carried out with a TAKARA PrimeScript™ RT reagent Kit with gDNA Eraser and TB Green® Premix Ex Taq™ II [51]. Expression levels were calculated by the 2^−ΔΔCt^ method [52] and normalized to the level of the reference gene *Actin7*.

### Statistical analysis

The data obtained were analysed using IBM SPSS Statistics 26 and are presented as the mean ± SE. Independent-samples T test was used for comparisons analysis.

## Supplementary Information

**Additional file 1: Figure S1.** Adaxial surface patterns of leaves. (a-b): the enlarged picture of Fig. 2D and E in the manuscript, both of which have clear trichomes. (c): the trichomes on the leaf, which are closely attached to the leaf epidermis cells and almost in the same focus as the epidermal cells. (d): adaxial surface patterns of variegated leaves under transmitted light. The white leaf variegation still has dark cell edges, while the green one does not. These evidences can prove that the observation regarding darker cellular edges in white type do exist. W: the white sector; G: the green sector. **Figure S2.** The length distribution of assembled unigenes. **Figure S3.** Cluster of DEGs. **Figure S4.** Volcano plot of DEGs. **Table S1.** An overview of the RNA-Seq data. **Table S2.** The primers for qRT-PCR analysis.

## Data Availability

The datasets generated and analysed in our study are available in the NCBI Sequence Read Archive under the accession number PRJNA815489.

## Abbreviations

DEGs: Differentially expressed genes
LRR-RLKs: Leucine-rich repeat receptor-like kinases
*XET/H*: Xyloglucan endotransglycosylase/hydrolase
PE: Pectinesterase
*EXP*: Expansin
*CSL*: Cellulose synthase-like
*VAR3*: *VARIEGATED 3*
*HEMC*: Hydroxymethylbilane synthase
*CHLH*: The H subunit of magnesium chelatase
*CHL2*: Chlorophyllase-2
*VDL*: *Variegated and Distorted Leaf*
*DCL*: *Defective Chloroplasts and Leaves*
GO: Gene Ontology
KEGG: Kyoto Encyclopedia of Genes and Genomes
SP: Spotted pattern
PP: Polygonal pattern
Chl: Chlorophyll
Chl a: Chlorophyll a
Chl b: Chlorophyll b
Chl * a+b*: Chlorophyll *a+b*
Chl * a/b*: the ratio of chlorophyll *a*/chlorophyll *b*
PS II: Photosystem II
Fv/Fm: Maximum photochemical quantum yield of PS
Y(PSII): Effective photochemical quantum yield of PS II
FW: Fresh weight
CC: Cellular component
MF: Molecular function
BP: Biological process
FAA: Formaldehyde-acetic acid-ethanol fixative

## References

[1] Hara N (1957). Study of the variegated leaves with special reference to those caused by air spaces. Jpn. J. Bot.

[2] Sheue CR, Pao SH, Chien LF, Chesson P, Peng CI (2012). Natural foliar variegation without costs? The case of Begonia. Ann. Bot..

[3] Burtt BL (1977). Notes on the rain-forest herbs. Gardens’ Bulletin, Singapore.

[4] Tsukaya H, Okada H, Mohamed M (2004). A novel feature of structural variegation in leaves of the tropical plant S*chismatoglottis calyptrata*. J. Plant Res.

[5] Zhang JH, Zeng JC, Wang XM, Chen SF, Albach DC, Li HQ (2020). A revised classification of leaf variegation types. Flora.

[6] Chen YS, Chesson P, Wu HW, Pao SH, Liu JW, Chien LF, Yong JWH, Sheue CR (2017). Leaf structure affects a plant’s appearance: combined multiple mechanisms intensify remarkable foliar variegation. J. Plant Res.

[7] Rocca NL, Rascio N, Pupillo P (2011). Variegation in *Arum italicum* leaves. A structural-functional study. Plant Physiol. Biochem.

[8] Stehlik I, Stinchcombe JR, Campitelli BE (2008). Leaf variegation is associated with reduced herbivore damage in *hydrophyllum virginianum*. Botany.

[9] Shelef O, Summerfield L, Lev-Yadun S, Villamarin-Cortez S, Sadeh R, Herrmann I, Rachmilevitch S (2019). Thermal benefits from white variegation of *Silybum marianum* leaves. Front. Plant Sci.

[10] Liu XY, Zheng MD, Wang R, Wang RJ, An LJ, Rodermel SR, Yu F (2013). Genetic interactions reveal that specific defects of chloroplast translation are associated with the suppression of *var2-Mediated* leaf variegation. J. Integr. Plant Biol..

[11] Sakamoto W (2003). Leaf-variegated mutations and their responsible genes in *Arabidopsis thaliana*. Genes Genet. Syst.

[12] Grimbly PE (1977). Tomato silvering, its anatomy and chimerical structure. J. Hortic. Sci..

[13] Ishizaki K (2015). Development of schizogenous intercellular spaces in plants. Front. Plant Sci.

[14] Ishizaki K, Mizutani M, Shimamuru M, Masuda A, Nishiama R, Kohchi T (2013). Essential role of the E3 Ubiquitin Ligase *NOPPERABO1* in schizogenous intercellular space formation in the liverwort *Marchantia polymorpha*. Plant Cell.

[15] Farrokhi N, Burton RA, Brownfield L, Hrmova M, Fincher GB (2006). Plant cell wall biosynthesis: genetic, biochemical and functional genomics approaches to the identification of key genes. Plant Biotechnol. J.

[16] Van Sandt VS, Suslov D, Verbelen JP, Vissenberg K (2007). Xyloglucan endotransglucosylase activity loosens a plant cell wall. Ann Bot.

[17] Phan TD, Bo W, West G, Lycett GW, Tucker GA (2007). Silencing of the major salt-dependent isoform of pectinesterase in tomato alters fruit softening. Plant Physiol.

[18] Marowa P, Ding A, Kong Y (2016). Expansins: roles in plant growth and potential applications in crop improvement. Plant Cell Rep.

[19] Park S, Szumlanski AL, Gu F, Guo F, Nielsen E (2011). A role for csld3 during cell-wall synthesis in apical plasma membranes of tip-growing root-hair cells. Nat. Cell Biol..

[20] Wang Y, Duby G, Purnelle B, Boutry M (2000). Tobacco *VDL* gene encodes a plastid DEAD box RNA helicase and is involved in chloroplast differentiation and plant morphogenesis. Plant Cell.

[21] Kwddie JS, Carrol B, Jone JDG, Gruissem W (1996). The DCL gene of tomato is required for chloroplast development and palisade cell morphogenesis in leaves. EMBO J..

[22] Naested H, Arabidopsis (2004). *VARIEGATED 3* encodes a chloroplast-targeted, zinc-finger protein required for chloroplast and palisade cell development. J. Cell Sci.

[23] Munekage YN, Inoue S, Yoneda Y, Yokota A (2015). Distinct palisade tissue development processes promoted by leaf autonomous signalling and long-distance signalling in *Arabidopsis thaliana*. Plant, Cell Environ..

[24] Camacho-Cristobal JJ, Herrera-Rodríguez MB, Beato VM, Rexach J, Navarro Gochicoa MT, Maldonado JM (2008). The expression of several cell wall-related genes in Arabidopsis roots is down-regulated under boron deficiency. Environ. Exp. Bot.

[25] Jensen PE, Reid JD, Hunter CN (2000). Modification of cysteine residues in the ChlI and ChlH subunits of magnesium chelatase results in enzyme inactivation. Biochem. J..

[26] Minoru K, Susumu G (2000). KEGG: Kyoto encyclopedia of genes and genomes. Nucleic Acids Res.

[27] Konoplyova A, Petropoulou Y, Yiotis C, Psaras GK, Manetas Y (2008). The fine structure and photosynthetic cost of structural leaf variegation. Flora.

[28] Rocca NL, Pupillo P, Puppi G, Rascio N (2013). Erythronium dens-canis l. (liliaceae): an unusual case of change of leaf mottling. Plant Physiol Biochem.

[29] Zhang Y, Hayashi T, Hosokawa M, Yazawa S, Li YH (2009). Metallic lustre and the optical mechanism generated from the leaf surface of *begonia rex* putz. Sci. Hortic.

[30] Fooshee WC, Henny RJ (1990). Chlorophyll leaves and anatomy of variegated and nonvarigated areas of *Aglaonema nitudum* leaves. Proc Fla State Hortic Soc.

[31] Li WX, Yang SB, Lu ZG, He ZC, Ye YL, Zhao BB, Wang L, Jin B (2018). Cytological, physiological, and transcriptomic analyses of golden leaf coloration in *Ginkgo biloba* L. Hortic. Res.

[32] Zhang Q, Huang J, Zhou P, Hao M, Zhang M (2021). Cytological and transcriptomic analysis provide insights into the formation of variegated leaves in *Ilex × altaclerensis* ‘Belgica Aurea’. Plants.

[33] Jinn TL, Stone JM, Walker JC (2000). *HAESA*, an *arabidopsis* leucine-rich repeat receptor kinase, controls floral organ abscission. Genes Dev.

[34] Harris PJ, McQueen-Mason S, Darley C, Roberts P, Jones L, Thomas B, Murray BG, Murphy DJ (2017). Cell Growth. Encyclopedia of Applied Plant Sciences.

[35] Yu F, Fu A, Aluru M, Park S, Xu Y, Liu H (2007). Variegation mutants and mechanisms of chloroplast biogenesis. Plant, Cell Environ.

[36] Gomez FM, Carrión CA, Costa ML, Desel C, Kieselbach T, Funk C, Krupinska K, Guiamét J. Extra-Plastidial degradation of chlorophyll and photosystem I in tobacco leaves involving ‘senescence-associated vacuoles.’ Plant J. 2019;99:465–77. 10.1111/tpj.14337. 10.1111/tpj.14337 30985038

[37] Walker CJ, Willows RD (1997). Mechanism and regulation of Mg-chelatase. Biochem. J..

[38] Papenbrock J, Mock HP, Tanaka R, Kruse E, Grimm B (2000). Role of magnesium chelatase activity in the early steps of the tetrapyrrole biosynthetic pathway. Plant Physiol.

[39] Zhu GF, Yang FX, Shi SS, Li DM, Wang Z, Liu HL, Dan H, Wang CY (2015). Transcriptome Characterization of *Cymbidium sinense* ‘Dharma’ Using 454 Pyrosequencing and Its Application in the Identification of Genes Associated with Leaf Color Variation. PLoS ONE.

[40] Wang F, Chen N, Shen S (2022). iTRAQ-Based Quantitative Proteomics Analysis Reveals the Mechanism of Golden-Yellow Leaf Mutant in Hybrid Paper Mulberry. Int. J. Mol. Sci.

[41] Pollard M, Beisson F, Li Y, Ohlrogge JB (2008). Building lipid barriers: biosynthesis of cutin and suberin. Trends Plant Sci.

[42] Hu Y, Yang L, Gao C, Liao DS, Long L, Qiu J, Wei HL, Deng QE, Zhou YC (2022). A comparative study on the leaf anatomical structure of *Camellia oleifera* in a low-hot valley area in Guizhou Province, China. PLoS ONE.

[43] Zhu JY, Xu JL, Cao YJ, Fu J, Li BL, Sun GP (2021). Leaf reflectance and functional traits as environmental indicators of urban dust deposition. BMC Plant Biol.

[44] Arnon DI (1949). Copper enzymes in isolated chloroplasts. polyphenoloxidase in *Beta Vulgaris*. Plant Physiol.

[45] Wan YL, Zhang YX, Zhang M, Hong AY, Yang HY, Liu Y (2020). Shade effects on growth, photosynthesis and chlorophyll fluorescence parameters of three *Paeonia* species. PeerJ.

[46] Demmig-Adams B, Adams WW, Barker DH, Logan BA, Bowling DR, Verhoeven AS (1996). Using chlorophyll fluorescence to assess the fraction of absorbed light allocated to thermal dissipation of excess excitation. Physiol. Plant..

[47] Hendrickson L, Furbank RT, Chow WS (2004). A simple alternative approach to assessing the fate of absorbed light energy using chlorophyll fluorescence. Photosynth. Res..

[48] Grabherr MG, Haas BJ, Yassour M, Levin JZ, Amit I (2011). Full-length transcriptome assembly from RNA-Seq data without a reference genome. Nat Biotechnol.

[49] Love MI, Huber W, Anders S (2014). Moderated estimation of fold change and dispersion for RNA-Seq data with DESeq2. Genome Biol.

[50] Wang J, Wang H, Ding L, Song A, Shen F, Jiang J (2017). Transcriptomic and hormone analyses reveal mechanisms underlying petal elongation in chrysanthemum morifolium ‘jinba’. Plant Mol. Biol.

[51] Livak KJ, Schmittgen TD (2001). Analysis of relative gene expression data using real-time quantitative PCR and the 2 – ∆∆CT Method. Methods.

[52] Li H, Meng H, Sun X, Deng J, Chen Q (2021). Integrated microRNA and transcriptome profiling reveal key miRNA-mRNA interaction pairs associated with seed development in Tartary buckwheat (Fagopyrum tataricum). BMC Plant Biol.

