# Supplementary material for: Cytological, physiological and transcriptomic analysis of variegated Leaves in Primulina pungentisepala offspring
Source: BMC Plant Biol. 2022 Sep 1;22:419. doi: 10.1186/s12870-022-03808-1 (PMC9434889; doi:10.1186/s12870-022-03808-1)
Supplement: Supplementary file 1 — Additional file 1: Figure S1. Adaxial surface patterns of leaves. (a-b): the enlarged picture of Fig. 2D and E in the manuscript, both of which have clear trichomes. (c): the trichomes on the leaf, which are closely attached to the leaf epidermis cells and almost in the same focus as the epidermal cells. (d): adaxial surface patterns of variegated leaves under transmitted light. The white leaf variegation still has dark cell edges, while the green one does not. These evidences can prove that the observation regarding darker cellular edges in white type do exist. W: the white sector; G: the green sector. Figure S2. The length distribution of assembled unigenes. Figure S3. Cluster of DEGs. Figure S4. Volcano plot of DEGs. Table S1. An overview of the RNA-Seq data. Table S2. The primers for qRT-PCR analysis. [file 12870_2022_3808_MOESM1_ESM.docx]

**Additional file 1**


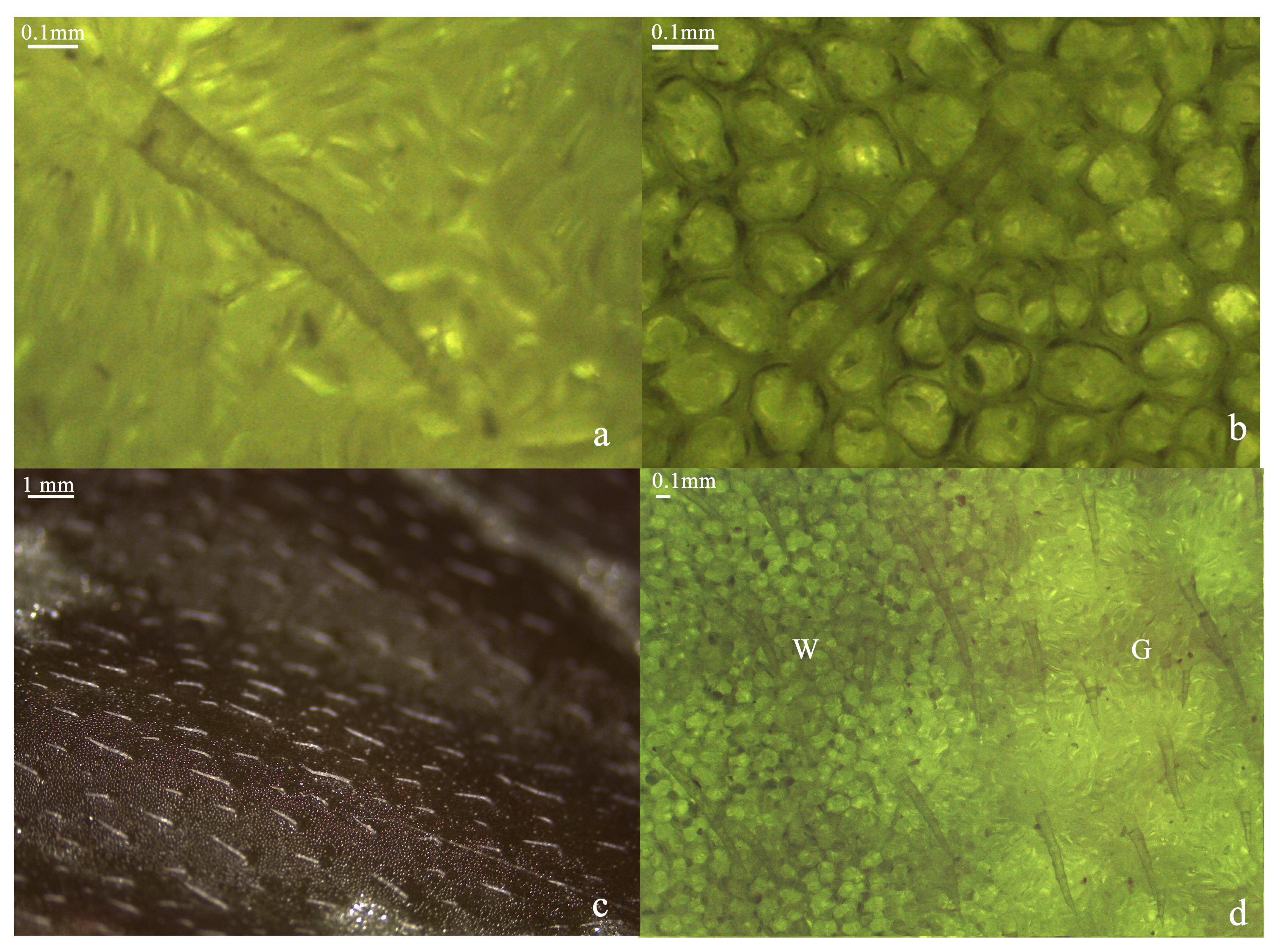


**Figure S1.** Adaxial surface patterns of leaves. (a-b): the enlarged picture of Fig. 2D and 2E in the manuscript, both of which have clear trichomes. (c): the trichomes on the leaf, which are closely attached to the leaf epidermis cells and almost in the same focus as the epidermal cells. (d): adaxial surface patterns of variegated leaves under transmitted light. The white leaf variegation still has dark cell edges, while the green one does not. These evidences can prove that the observation regarding darker cellular edges in white type do exist. W: the white sector; G: the green sector.





**Figure S2**. The length distribution of assembled unigenes.


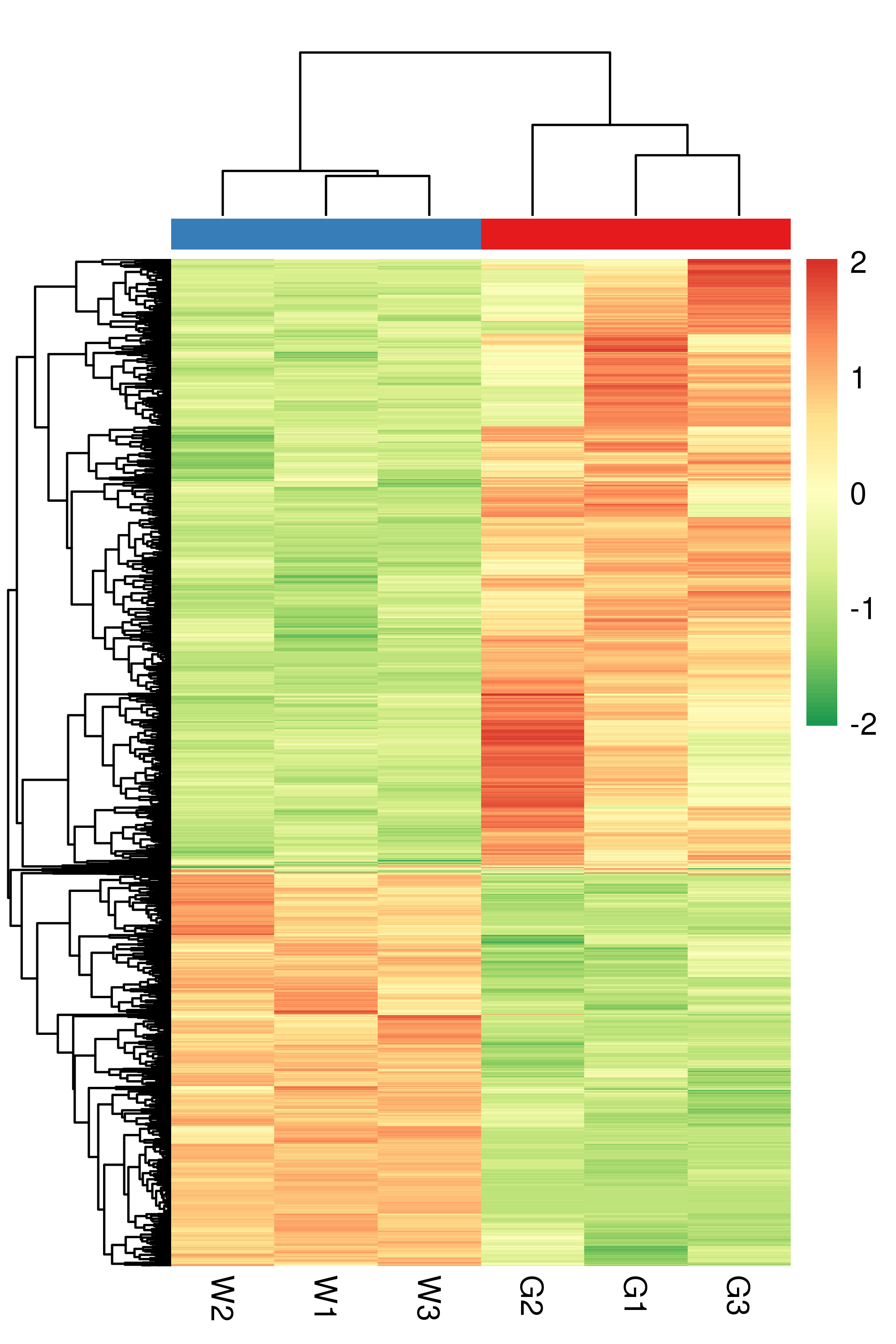


**Figure S3.** Cluster of DEGs


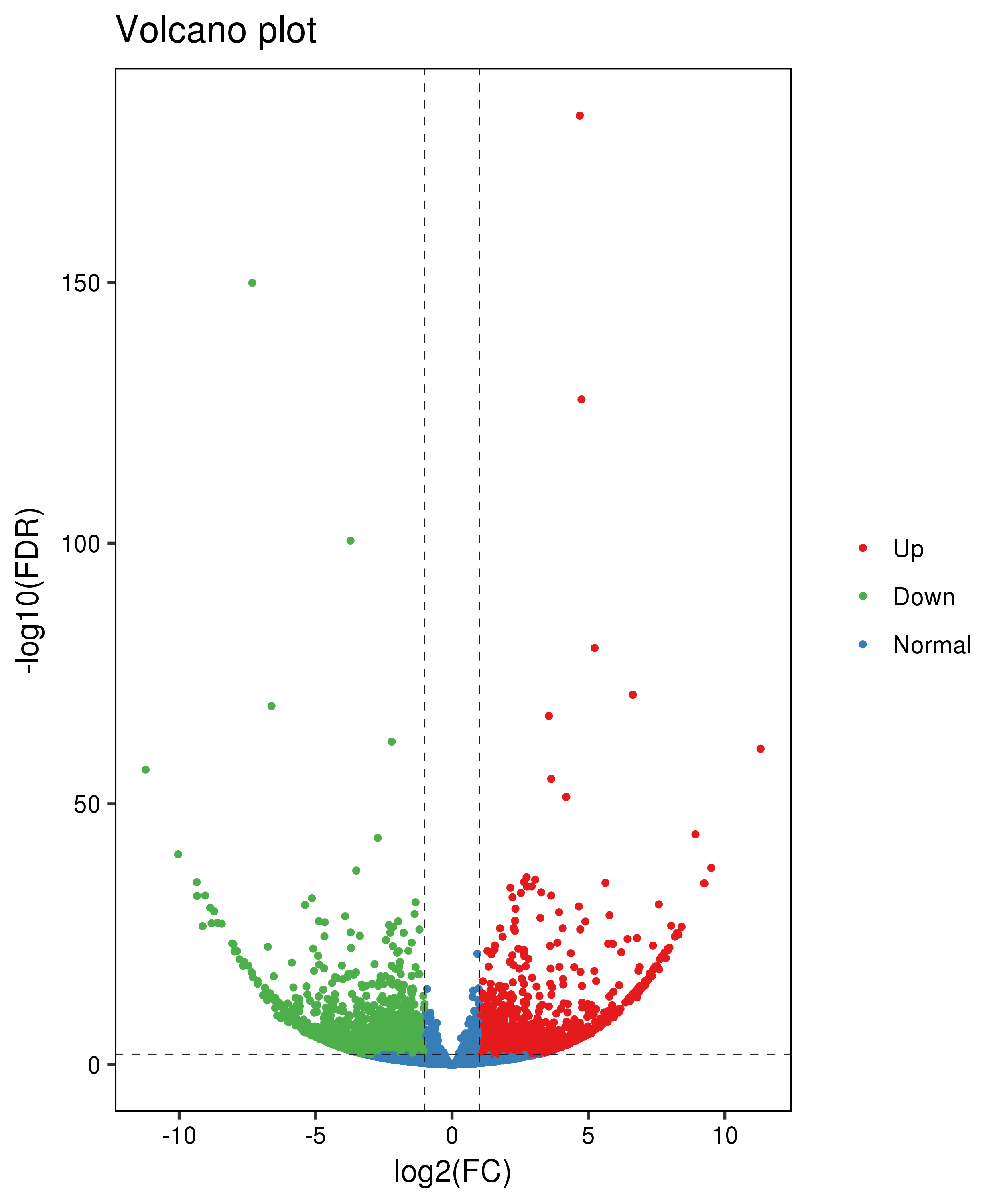


**Figure S4.** Volcano plot of DEGs

**Table S1.** An overview of the RNA-Seq data

| **Sample** | **Raw Reads** | **Clean Reads** | **Raw Base(G)** | **Clean Base(G)** | **Effective Rate(%)** | **Error Rate(%)** | **Q20(%)** | **Q30(%)** | **GC Content(%)** | **Mapped Reads** | **Mapped Ratio(%)** |
| --- | --- | --- | --- | --- | --- | --- | --- | --- | --- | --- | --- |
| G1 | 23 616 335 | 22 560 312 | 7.08 | 6.77 | 95.53 | 0.03 | 97.43 | 92.61 | 44.37 | 14060071 | 68.82 |
| G2 | 23 813 002 | 22 883 384 | 7.14 | 6.87 | 96.1 | 0.03 | 97.78 | 93.55 | 44.99 | 15808565 | 70.07 |
| G3 | 21 229 962 | 20 430 288 | 6.37 | 6.13 | 96.23 | 0.03 | 97.6 | 93.16 | 45.17 | 16066032 | 70.21 |
| W1 | 19 757 500 | 18 652 487 | 5.93 | 5.6 | 94.41 | 0.03 | 97.42 | 92.67 | 44.7 | 13661218 | 67.68 |
| W2 | 21 125 523 | 19 818 691 | 6.34 | 5.95 | 93.81 | 0.03 | 97.69 | 93.33 | 44.56 | 13588566 | 68.56 |
| W3 | 20 883 408 | 19 847 115 | 6.27 | 5.95 | 95.04 | 0.03 | 97.72 | 93.33 | 44.53 | 13431085 | 67.67 |

**Table S2.** The primers for qRT-PCR analysis.

| **DEGs** | **Primer sequences** |
| --- | --- |
| TRINITY_DN6903_c0_g1-F | TTGAGTGCCAACCATCTCC |
| TRINITY_DN6903_c0_g1-R | TTGAAAGGGAACAAAGGAAG |
| TRINITY_DN8967_c0_g1-F | GAACCTAACTTTCGTGGACAT |
| TRINITY_DN8967_c0_g1-R | CGGAGGCTGAACTGCTAAT |
| TRINITY_DN27781_c0_g1-F | TGATGGGTGCTGTTGAGGG |
| TRINITY_DN27781_c0_g1-R | CGAAGAACCCGAACATTGAG |
| TRINITY_DN7086_c0_g1-F | TCCGATTTATGAAGCAGAGC |
| TRINITY_DN7086_c0_g1-R | TCCTAGCCCCAAAGCAGAC |
| TRINITY_DN6080_c0_g1-F | CTAAGTTCAGACGGACGGC |
| TRINITY_DN6080_c0_g1-R | CCTCACTGGATACTATGTTTTGC |
| TRINITY_DN7812_c0_g1-F | AGTAATGTTCCCCTCTTTTCAC |
| TRINITY_DN7812_c0_g1-R | TACCTTGGCTGTTGTCTCG |
| TRINITY_DN13428_c0_g1-F | TCACTTCTTTCGGAACCACG |
| TRINITY_DN13428_c0_g1-R | GGTGAATCCGCAGTGTAAGA |
| TRINITY_DN6896_c1_g1-F | GTTGAGGGCTACCGTATTGC |
| TRINITY_DN6896_c1_g1-R | TCAACTCAGCGAATGCCTC |
| TRINITY_DN17252_c0_g1-F | GCGATTACCGTGTTCAGTT |
| TRINITY_DN17252_c0_g1-R | GTTAAGATTCGGGCTGTTG |
| TRINITY_DN511_c0_g1-F | AAGCAGTAAAACCCACGAGT |
| TRINITY_DN511_c0_g1-R | CAAATCGAAGTCCCTGTGAG |
| Actin7-F | AACCACCGCTGAGCACGAT |
| Actin7-R | TTCCGTTGCCCTGAAGTCC |
